# d‐amino acid oxidase knockout (*Dao* ^−/−^) mice show enhanced short‐term memory performance and heightened anxiety, but no sleep or circadian rhythm disruption

**DOI:** 10.1111/ejn.12880

**Published:** 2015-03-27

**Authors:** David Pritchett, Sibah Hasan, Shu K. E. Tam, Sandra J. Engle, Nicholas J. Brandon, Trevor Sharp, Russell G. Foster, Paul J. Harrison, David M. Bannerman, Stuart N. Peirson

**Affiliations:** ^1^ Nuffield Department of Clinical Neurosciences (Nuffield Laboratory of Ophthalmology)John Radcliffe HospitalUniversity of OxfordOxfordOX3 9DUUK; ^2^ Pfizer Inc.GrotonCTUSA; ^3^ Department of PharmacologyUniversity of OxfordOxfordUK; ^4^ Department of PsychiatryWarneford HospitalUniversity of OxfordOxfordUK; ^5^ Department of Experimental PsychologyUniversity of OxfordSouth Parks RoadOxfordOX1 3UDUK; ^6^ AstraZeneca Neuroscience iMEDCambridgeMA02139USA

**Keywords:** d‐serine, glutamate, *N*‐methyl‐d‐aspartate receptor, preclinical model, schizophrenia

## Abstract

d‐amino acid oxidase (DAO, DAAO) is an enzyme that degrades d‐serine, the primary endogenous co‐agonist of the synaptic *N*‐methyl‐d‐aspartate receptor. Convergent evidence implicates DAO in the pathophysiology and potential treatment of schizophrenia. To better understand the functional role of DAO, we characterized the behaviour of the first genetically engineered *Dao* knockout (*Dao* ^−/−^) mouse. Our primary objective was to assess both spatial and non‐spatial short‐term memory performance. Relative to wildtype (*Dao* ^+/+^) littermate controls, *Dao* ^−/−^ mice demonstrated enhanced spatial recognition memory performance, improved odour recognition memory performance, and enhanced spontaneous alternation in the T‐maze. In addition, *Dao* ^−/−^ mice displayed increased anxiety‐like behaviour in five tests of approach/avoidance conflict: the open field test, elevated plus maze, successive alleys, light/dark box and novelty‐suppressed feeding. Despite evidence of a reciprocal relationship between anxiety and sleep and circadian function in rodents, we found no evidence of sleep or circadian rhythm disruption in *Dao* ^−/−^ mice. Overall, our observations are consistent with, and extend, findings in the natural mutant ddY/*Dao* ^−^ line. These data add to a growing body of preclinical evidence linking the inhibition, inactivation or deletion of DAO with enhanced cognitive performance. Our results have implications for the development of DAO inhibitors as therapeutic agents.

## Introduction

d‐amino acid oxidase (DAO, DAAO, EC 1.4.3.3) is a peroxisomal enzyme that catalyses the degradation of neutral d‐amino acids such as d‐serine (Pollegioni *et al*., 2007). Through its action at the glycine site, d‐serine is a potent endogenous co‐agonist of the synaptic *N*‐methyl‐d‐aspartate receptor (NMDAR) (Schell *et al*., 1995; Mothet *et al*., 2000; Oliet & Mothet, 2009; Papouin *et al*., 2012). Hence, DAO is able to influence NMDAR function by regulating the availability of d‐serine within synapses (Schell *et al*., 1997; Mothet *et al*., 2000; Almond *et al*., 2006; Panatier *et al*., 2006). On this basis, DAO is potentially relevant to neurological or psychiatric disorders in which NMDAR function is abnormal. For example, *Dao* expression and DAO activity are increased in schizophrenia (Kapoor *et al*., 2006; Verrall *et al*., 2007; Burnet *et al*., 2008; Madeira *et al*., 2008; Habl *et al*., 2009; Ono *et al*., 2009), which may contribute to the NMDAR hypofunction thought to occur in the disorder (Olney *et al*., 1999; Kantrowitz & Javitt, 2010; Marek *et al*., 2010; Verrall *et al*., 2010; Coyle, 2012; Labrie *et al*., 2012). DAO inhibitors could therefore prove useful in the treatment of schizophrenia (Smith *et al*., 2010; Ferraris & Tsukamoto, 2011; Sacchi *et al*., 2013).

To explore the functional role of DAO, behaviour has been assessed in two related mutant mouse lines that lack DAO activity, and in wildtype rodents following the administration of d‐serine or DAO inhibitors. These models demonstrate enhanced performance in several long‐term memory paradigms, including the Barnes maze, Morris watermaze (MWM), and object recognition memory tests with long retention intervals (Maekawa *et al*., 2005; Duffy *et al*., 2008; Hashimoto *et al*., 2008; Karasawa *et al*., 2008; Labrie *et al*., 2009b; Smith *et al*., 2009; Bado *et al*., 2011; Zhang *et al*., 2011; Hopkins *et al*., 2013). Although T‐maze rewarded alternation is enhanced in wildtype mice following d‐serine administration (Bado *et al*., 2011), short‐term memory has yet to be assessed in a genetic mouse model lacking DAO activity. A single nucleotide polymorphism in *Dao* is associated with altered working memory performance in healthy men (Roussos *et al*., 2011), which hints at a role for DAO in short‐term memory. In addition to their cognitive phenotype, *Dao* mutant mice demonstrate a heightened anxiety phenotype, as revealed in both the elevated plus maze and open field test (Labrie *et al*., 2009a). Likewise, wildtype mice display increased anxiety‐like behaviour in the elevated plus maze after d‐serine administration (Labrie *et al*., 2009a).

To confirm and expand upon these data, we characterized the behaviour of the first genetically engineered *Dao* knockout (*Dao* ^−/−^) mouse. Our assessment of *Dao* ^−/−^ mice in the MWM revealed a complex behavioural phenotype, which is documented in detail in a forthcoming article (D. Pritchett, A.M. Taylor, S.J. Engle, N.J. Brandon, T. Sharp, R.G. Foster, P.J. Harrison, S.N. Peirson and D.M. Bannerman, in preparation). In the present study, *Dao* ^−/−^ mice were subjected to tests of spatial and non‐spatial *short‐term* memory, and we also sought to establish whether *Dao* ^−/−^ mice (like *Dao* mutants) display an elevated anxiety phenotype. Lastly, we characterized sleep and circadian rhythms in *Dao* ^−/−^ mice, given that: (i) there is evidence of a bidirectional relationship between anxiety and sleep and circadian function in rodents (Silva *et al*., 2004; Koehl *et al*., 2006; Tang *et al*., 2007; Pawlyk *et al*., 2008; Jakubcakova *et al*., 2012; Griesauer *et al*., 2014); (ii) NMDAR antagonists block the circadian effects of light in wildtype mice (Colwell *et al*., 1991); and (iii) DAO has an established physiological role in the retina (Stevens *et al*., 2003; Gustafson *et al*., 2007, 2013), which provides light information to the primary circadian pacemaker in the suprachiasmatic nucleus of the hypothalamus (Moore, 1973; Moore & Klein, 1974).

## Materials and methods

### Animals

The *Dao* knockout mice were generated as described previously (Rais *et al*., 2012). Briefly, recombineering was used to replace 1957 bp of *Dao* (encompassing exons 7 and 8) with a neomycin cassette, and the mutant allele was introduced into 129SvEv mouse embryonic stem cells (TG‐ES01‐01 ESM07; Eurogentec, Seraing, Belgium) by standard homologous recombination. Male chimeric mice were generated by injecting the targeted embryonic stem cells into C57BL/6J blastocysts. Chimeric mice were bred with 129SvEv mice (Taconic, Hudson, NY, USA) to produce F_1_ heterozygotes, which in turn were mated to produce *Dao* knockout (*Dao* ^−/−^) mice and wildtype (*Dao* ^+/+^) littermate controls.

Mice were at least 2 months old at the onset of all procedures, and no older than 9 months upon completion of testing. A total of seven cohorts were used, full details of which are provided in Table S1. Males and females were subjected to behavioural testing, but only males participated in the sleep and circadian screen due to the potentially confounding influence of the oestrus cycle on wheel‐running activity (e.g. Pilorz *et al*., 2009). During behavioural testing, mice were housed under a 100 lux 12 : 12 h light/dark (12 : 12 LD) cycle, with access to food and water *ad libitum*. All behavioural procedures were performed in accordance with the United Kingdom Animals (Scientific Procedures) Act of 1986 and the University of Oxford Policy on the Use of Animals in Scientific Research. All experiments were approved by the University of Oxford Animal Welfare and Ethical Review Board, and were conducted under the project licences (PPLs) 30/2812 and 30/3068 by the personal licence holders (PILs) ICC0614BD, I459D3D59 and I869292DB. Upon completion of all procedures, mice were culled by cervical dislocation, and death was confirmed by the onset of rigor mortis.

### General behavioural protocol

Prior to behavioural testing, mice were handled for 2 min/day for five consecutive days to habituate them to experimenter handling. For all experiments, mice were brought into the test room 5–10 min prior to the onset of testing. The test apparatus was cleaned with diluted ethanol between trials. Illuminance was 50 lux at the base of each apparatus, unless otherwise stated. With the exception of T‐maze spontaneous alternation, novelty‐suppressed feeding and spontaneous locomotor activity, all behavioural experiments were recorded with a webcam (Logitech, Morges, Switzerland) or a near‐infrared CCTV camera (Maplin Electronics, Rotherham, UK). Automated tracking was conducted using any‐maze 4.5 (Stoelting, Wood Dale, IL, USA). The animal's entire body was tracked, and its position within the apparatus was determined on a frame‐by‐frame basis. An entry to a predefined zone was deemed to have occurred when at least 90% of the animal's body had entered the zone in question.

### Spontaneous locomotor activity

Mice were transferred from their home‐cage to a novel, open‐top cage (42 cm long × 22 cm wide × 20 cm high), resting within a photobeam frame (San Diego Instruments, San Diego, CA, USA). Locomotor activity was quantified as the number of beam breaks over a 2 h period, divided into twenty‐four 5 min time bins. This interval was selected to enable direct comparison with the anxiety tests, four of which lasted 5 min.

### Short‐term memory

#### Spontaneous recognition memory

**Figure 1:**
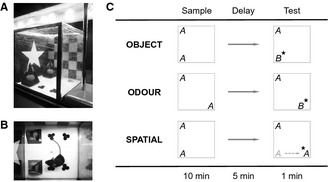
Apparatus and protocol for testing spontaneous recognition memory. (A, B) Side and top views of the apparatus, respectively. Patterned wallpapers were attached to two walls, to facilitate discrimination of the four corners of the arena. (C) A schematic showing the different versions of the spontaneous recognition task. In object and odour recognition trials, the mouse explored two identical replicates of an object or odour cue ‘A’ for 10 min during the sample phase. After a 5 min delay (denoted by →), one replicate of ‘A’ was replaced by a novel object or odour cue ‘B’, and the animal was allowed to explore ‘A’ and ‘B’ for 1 min during the test phase. In spatial recognition trials, no novel object was introduced. Instead, one replicate of ‘A’ was displaced to a novel location. For each trial type, the star denotes the novel stimulus in the test phase.

Spontaneous recognition memory testing took place in an open‐top arena (20 × 20 × 20 cm), made of transparent acrylic (Fig. 1A and B). To facilitate discrimination of the four corners, patterned wallpapers were attached to the outside of two walls. Velcro was attached to the base of the arena and the bottom of all experimental stimuli, so that they could be fixed in place during behavioural testing. Illumination was 200 lux at the base of the apparatus. All mice completed two habituation trials (on separate days) prior to testing. In these trials, mice were able to explore the empty apparatus for 9 min. Subsequently, all mice completed two trials of object recognition memory testing, two trials of spatial recognition memory testing, and two trials of odour recognition memory testing. Each trial was performed on a separate day, and the six trials were presented in a pseudo‐randomized order, which was counterbalanced across genotype groups. Experimental trials consisted of a sample phase (10 min), a delay period (5 min), and a test phase (1 min).

In object recognition trials, mice explored two identical replicates of an object during the sample phase. During the delay period, the animal was removed from the arena and placed in an empty holding cage, while the arena was cleaned with diluted ethanol. One replicate was replaced by an entirely novel object, while the other was replaced by a third replicate of the familiar object (Fig. 1C). Multiple replicates were employed to prevent mice from re‐encountering their own odour cues. Odour recognition trials were similar to object recognition trials, except that the stimulus was not a household object but 1 mL of food flavouring (Dr. Oetker, Bielefeld, Germany) inside a transparent shot‐glass. The to‐be‐discriminated object/odour pairings have been validated previously in our laboratory. Object pairings differed along multiple sensory dimensions (i.e. colour, size, shape, and texture). For each pairing, the identity of the novel object and its location at test (i.e. top or bottom) were counterbalanced across genotype groups. In spatial recognition trials, a novel object was not presented in the test phase. Instead, the test phase involved exposure to two replicates of the familiar object. One remained in the same location as in the sample phase, whereas the other was displaced to a novel location (Fig. 1C). The location selected for displacement (i.e. top or bottom) was counterbalanced across genotype groups.

The total distance travelled during the habituation, sample and test phases was calculated using any‐maze. The amount of time spent in contact with the experimental stimuli (during the sample and test phases) was derived manually from the videos by an observer who was blind to both the genotype of the animals and the identity and location of the novel stimulus. Recognition memory performance in each trial was expressed as a ratio, *n*/(*n* + *f*), where *f* and *n* represent the total amount of time spent in contact with the familiar and novel stimuli during the test phase. One‐sample *t*‐tests (two‐tailed) were used to compare recognition ratios against chance performance (0.5).

#### T‐maze spontaneous alternation

The apparatus was a black wooden T‐maze. The walls were 21 cm high, while each of the arms was 30 cm long and 9 cm wide. A removable central partition (extending 14 cm into the start arm) was used during the sample phase but not the test phase of each trial. Guillotine doors were positioned at the entrance to each goal arm. At the beginning of the sample phase, both doors were raised, and the mouse was placed at the end of the start arm facing away from the goal arms. Each mouse was allowed to make a free choice between the two goal arms; after its tail had cleared the door of the chosen arm, the door was closed, and the mouse was allowed to explore the arm for 30 s. The mouse was then returned to the end of the start arm, with the central partition removed and both guillotine doors raised, signalling the beginning of the test phase. Again the mouse was allowed to make a free choice between the two goal arms. Trials that were not completed within 90 s were terminated and disregarded during analysis. In such cases, mice were returned to their home‐cage and retested after an interval of at least 10 min. All mice completed two trials per day over three consecutive days, with an intertrial interval of 4 h. The dependent variable was the percentage of trials in which mice successfully alternated between the sample and test phases. One‐sample *t*‐tests (two‐tailed) were used to compare these scores against chance performance (50%). Trial completion was also recorded (i.e. the proportion of trials completed within the 90 s cut‐off at the first attempt).

### Anxiety

#### Open field test

The apparatus was a rectangular arena measuring 60 × 30 cm, enclosed by four black walls, 25 cm in height. The base of the arena was a sheet of transparent acrylic, placed over a sheet of white card. Mice were released at the edge of the arena, facing towards the wall, and allowed to explore the apparatus for 5 min. Using any‐maze, the apparatus was divided into nominal peripheral and central zones (central zone: 46.5 cm long × 16.5 cm wide). The key variables extracted were centre entries, latency to the first centre entry, percent time spent in the centre, percent distance travelled in the centre, and total distance travelled.

#### Elevated plus maze

The apparatus was a white wooden plus maze, elevated 72 cm above the floor. All four arms were 35 cm long and 6 cm wide. The two closed arms were enclosed by grey walls, 21 cm in height. The mouse was placed in the centre of the maze, so that its head and forepaws fell within one of the open arms. Mice were allowed to explore the arena for 5 min. The key variables extracted were open‐arm entries, closed‐arm entries, percent time spent in the open arms, latency to the first open‐arm entry, and total distance travelled.

#### Successive alleys

The successive alleys test is an approach/avoidance task that can be conceptualized as a modified version of the elevated plus maze, which, like the plus maze, is sensitive to benzodiazepine administration (McHugh *et al*., 2004; Line *et al*., 2011; Deacon, 2013). The apparatus consisted of four linearly connected wooden alleys or ‘zones’, each 25 cm in length. The four zones differed in their physical characteristics (e.g. stability, width and colour), rendering zone 1 the least anxiogenic, and zone 4 the most anxiogenic (see Deacon, 2013). The apparatus was elevated 50 cm above the floor. Mice were released from the enclosed end of zone 1, facing the back wall, and were allowed to explore the apparatus for 5 min. The key variables extracted were the amount of time spent in (and entries to) each of the four zones, in addition to total distance travelled.

#### Light/dark box

The apparatus consisted of two identically‐sized compartments, each 30 cm long, 15 cm wide and 30 cm high, connected by a small opening (4.5 cm wide and 3.5 cm high). One compartment was made of opaque black acrylic, and the other was made of transparent acrylic. The walls of the transparent compartment were covered on the outside with white card. Mice were released in the centre of the light compartment, facing away from the opening, and were allowed to explore the arena for 5 min. The key variables extracted were percent time spent in the light compartment, number of light/dark crossings, and latency to the first light/dark crossing. Note that two mice were excluded from all light/dark box analyses as they froze for the entire trial and did not make any light/dark crossings (the average number of crossings amongst the remaining animals was 14.9).

#### Novelty‐suppressed feeding

All mice completed two trials, on separate days. Mice were partially food‐restricted for the entirety of the preceding dark phase; all food was removed from the food hopper and 1 g of standard chow was placed on the home‐cage floor. Testing began 1 h into the light phase. In the first trial, the novel food was a sucrose pellet, and the novel environment was the enclosed arm (25 cm long) of a black wooden Y‐maze, elevated 80 cm above the floor, with 0.5 cm high walls. In the second trial, the novel food was a sweetcorn niblet, and the novel environment was an unstable circular platform made of red acrylic (38 cm diameter), raised 64 cm above the floor, with no walls. Latency to first contact with the food was measured manually with a stopwatch, as was absolute latency to begin feeding. Feeding was defined as at least 2 s of continuous nibbling with the food held in the forepaws. Prior to analysis, latency to first contact with the food was subtracted from absolute latency to begin feeding, to control for differences in locomotor activity. Mice that did not feed within 2 min were removed from the apparatus and retested after 3 min. Each mouse was given a maximum of three attempts to feed, and a cumulative feeding latency was calculated, resulting in a maximum possible latency of 6 min.

### Circadian rhythms and sleep

#### Wheel‐running (circadian) analyses

This screen was based on an established protocol (Albrecht & Foster, 2002; Jud *et al*., 2005; Oliver *et al*., 2012). Briefly, mice were individually housed in large cages (44 cm long × 26 cm wide × 12 cm high) fitted with running wheels (18 cm diameter). Activity levels (i.e. wheel rotations) were recorded with the software package clocklab (Actimetrics, IL, USA). Cages were kept inside light‐tight chambers illuminated by an overhead cool white LED light source; illuminance was 100 lux at the base of each cage. Only cohorts C1 and C2 were tested (see Supporting Information). Cohort C1 was exposed to the following light schedules: 14 days of 12 : 12 LD, a 6 h phase advance during 12 : 12 LD, 11 days of constant dark (DD), and 7 days of constant light (LL). Cohort C2 was exposed to 12 : 12 LD only. Standard circadian parameters (see Supporting Information) were extracted from the raw wheel‐running data using the clocklab toolbox (Actimetrics) for matlab (MathWorks, MA, USA). Cohort C1 was also subjected to a type II phase‐shifting light pulse (Aschoff, 1965) from zeitgeber time (ZT)16 to ZT17 [using a previously‐described protocol (Albrecht *et al*., 2001; Jud *et al*., 2005)] to induce a phase delay in activity rhythms, providing an indicator of photosensitivity (ZT0 refers to the onset of the light phase, and ZT12 denotes the onset of the dark phase). Negative masking (the degree of activity suppression induced during the light pulse) was also calculated, yielding an additional measure of photosensitivity (Mrosovsky, 1999). Further details of these protocols are provided in the Supporting Information.

#### Video‐tracking (sleep) analyses

Video‐tracking analyses were based on an established protocol (Fisher *et al*., 2012). Only cohorts C1 and C2 were tested. At the end of the wheel‐running screen, mice were entrained to a 100 lux 12 : 12 LD cycle for 14 days and then transferred to identically‐sized cages (without running wheels) in new light‐tight chambers. A single 12 : 12 LD cycle was recorded with a near‐infrared CCTV camera (Maplin Electronics), at 3 frames/s, in the AVI file format. Recording began within 48 h of the transfer to the new cage, and an acrylic block was placed under the food hopper to keep the mouse in the recording field at all times. Video files were stored on a digital hard drive recorder (Samsung, Suwon, South Korea) prior to analysis with any‐maze 4.5 (Stoelting). Three parameters were extracted from the video footage: immobility‐determined sleep bouts, immobility‐determined sleep time, and distance travelled. Sleep time is expressed as a percentage of recording time. A sleep bout was defined as a period of immobility of at least 40 s, a previously established proxy measure of sleep (Fisher *et al*., 2012). This measure has an extremely high concordance (> 95%) with electroencephalography (EEG)‐based sleep determination (Fisher *et al*., 2012). Immobility sensitivity was set at 95% to prevent the detection of movements caused by breathing during sleep.

#### Electroencephalography‐based sleep analyses

As immobility‐determined sleep analyses do not distinguish rapid eye movement (REM) and non‐REM (NREM) sleep, EEG‐based sleep analyses were performed in an additional cohort of mice (cohort D). Six adult male mice (*Dao* ^+/+^, *n *=* *3; *Dao* ^−/−^, *n *=* *3; 3–4 months old) were implanted with a telemetric transmitter (volume 1.9 cm^3^; total weight 3.9 g; TL11M2‐F20‐EET; DSI, St Paul, MN, USA) connected to EEG and electromyography (EMG) electrodes, as described previously (Hasan *et al*., 2014). Surgery was performed under anaesthesia (isoflurane – induction 4.5%; maintenance 0.7–2.25%). Two stainless‐steel EEG electrodes (screw shaft length 2.4 mm; screw thread outer diameter 1.19 mm) were implanted epidurally over the right frontal and parietal cortices (Franken *et al*., 1998), and connected to the telemetric transmitter with medical‐grade stainless‐steel wires encased in silicone tubing. The EEG electrodes and connections to the subcutaneous wiring were covered with dental cement (RelyX Arc; Kent Express, Kent, UK). Two stainless‐steel EMG leads were inserted into the neck muscle ~5 mm apart and sutured in place. The telemetric transmitter was inserted into the peritoneal cavity. Perioperative analgesics were administered at the onset of surgery (buprenorphine, 0.1 mg/kg; meloxicam, 5 mg/kg) and on the following day (meloxicam, 5 mg/kg). Saline (0.9%; 500 μL) was administered by subcutaneous injection immediately after surgery. Animals were given 12–15 days to recover after surgery, and the telemetric transmitters were activated 2 days prior to data collection. EEG/EMG signals were recorded continuously for 24 h, starting at light onset (ZT0), using the software package dataquest ART (DSI). The resulting data were analysed using the software package sleepsign (Kissei Comtec, Nagano, Japan). The EEG and EMG signals were filtered with high‐pass (3 dB, 1.0 Hz) and low‐pass anti‐aliasing (49.5 Hz) analog filters. Wakefulness, REM sleep and NREM sleep were visually classified according to standard criteria (Franken *et al*., 1998), in 4 s epochs, without knowledge of the animal's genotype. Three parameters were extracted from the EEG/EMG data: total sleep time, REM sleep time, and NREM sleep time (all three are expressed as a percentage of recording time).

### Statistical analyses

All statistical analyses were performed with spss 22.0 (IBM, Armonk, New York, NY, USA). Unless otherwise stated, all reported statistics are the result of anovas, with genotype and sex included as independent variables, in addition to trial, day, stimulus type and time bin where applicable. Differences were considered to be statistically significant at *P*‐values < 0.05. Greenhouse–Geisser corrections were applied where appropriate, but uncorrected degrees of freedom are reported in order to preserve the transparency of the statistical design. For experiments involving multiple cohorts, further anovas were conducted with cohort included as an extra independent variable. No additional effects or interactions were observed, so these data are not shown. Defecation and urination were measured in all anxiety and memory experiments, but no effects were observed (data not shown).

## Results

### Spontaneous locomotor activity

Spontaneous locomotor activity was unaltered in *Dao* ^−/−^ mice (Fig. S1). Genotype had no effect on beam breaks during the first 5 min (*F*_1,20_ = 1.462, *P *=* *0.241), or the entire 2 h recording period (*F*_1,20_ = 0.022, *P* = 0.884). With the 2 h session divided into twenty‐four 5 min time bins, there was a main effect of time bin on beam breaks (*F*_23,460_ = 57.229, *P* < 0.001), but no genotype‐by‐time bin interaction (*F*_23,460_ = 0.790, *P* = 0.746), indicating that activity levels declined in *Dao* ^−/−^ and *Dao* ^+/+^ mice at a similar rate.

### Short‐term memory

#### Spontaneous recognition memory

**Figure 2:**
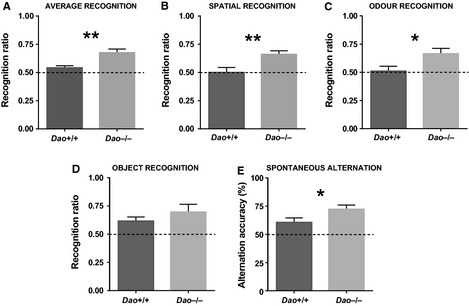
Evidence of enhanced short‐term memory performance in *Dao* ^−/−^ mice. (A) Average recognition memory performance was enhanced in *Dao* ^−/−^ mice relative to *Dao* ^+/+^ mice. (B) Spatial recognition memory performance was enhanced in *Dao* ^−/−^ mice. (C) Odour recognition memory performance was enhanced in *Dao* ^−/−^ mice. (D) Genotype had no significant effect on object recognition memory performance. (E) Spontaneous alternation in the T‐maze was enhanced in *Dao* ^−/−^ mice relative to *Dao* ^+/+^ mice. Dashed lines represent chance performance. *P ≤ 0.05, **P ≤ 0.01. Error bars depict the SEM.

Recognition ratios were entered into a repeated‐measures anova, with genotype as the principal between‐subjects variable, and stimulus type (i.e. object, odour or spatial) as a within‐subjects variable. This analysis yielded a main effect of genotype (*F*_1,19_ = 11.394, *P *=* *0.003; Fig. 2A), reflecting superior spontaneous recognition memory performance in *Dao* ^−/−^ mice. This enhancement was independent of stimulus type, as there was no interaction between genotype and stimulus type (*F*_2,38_ = 0.656, *P *=* *0.525). Likewise, there was no main effect of stimulus type (*F*_2,38_ = 1.387, *P *=* *0.262). With data collapsed across all three stimulus types, performance was above chance level (0.5) in *Dao* ^−/−^ mice (Student's *t*‐test, *t*_10_ = 5.324, *P *<* *0.001), but in *Dao* ^+/+^ mice, this difference was only borderline‐significant (Student's *t*‐test, *t*_11_ = 2.189, *P *=* *0.051).

Subsequently, *post hoc* anovas were conducted to assess recognition ratios for each stimulus type in isolation. Relative to *Dao* ^+/+^ mice, *Dao* ^−/−^ mice showed improved spatial recognition memory performance (*F*_1,19_ = 8.968, *P *=* *0.007, Fig. 2B) and odour recognition memory performance (*F*_1,19_ = 5.584, *P *=* *0.029, Fig. 2C). Object recognition memory performance was numerically but not significantly enhanced (*F*_1,19_ = 0.763, *P *=* *0.393, Fig. 2D). The effect of genotype on recognition memory performance is attributable to the fact that, during the test phase, *Dao* ^−/−^ mice spent more time than *Dao* ^+/+^ mice in contact with the novel stimulus, and less time than *Dao* ^+/+^ mice in contact with the familiar stimulus (Fig. S2).

In accordance with the results of these anovas, a series of one‐sample *t*‐tests confirmed that recognition ratios were generally poorer in *Dao* ^+/+^ mice. *Dao* ^+/+^ mice did not perform above chance in either odour (Student's *t*‐test, *t*_11_ = 0.212, *P *=* *0.836) or spatial (Student's *t*‐test, *t*_11_ = −0.059, *P *=* *0.954) trials, whereas *Dao* ^−/−^ mice performed above chance in both odour (Student's *t*‐test, *t*_10_ = 3.444, *P *=* *0.006) and spatial (Student's *t*‐test, *t*_10_ = 4.898, *P *=* *0.001) trials. In object trials, however, the performance of both *Dao* ^+/+^ mice (Student's *t*‐test, *t*_11_ = 3.045, *P *=* *0.011) and *Dao* ^−/−^ mice (Student's *t*‐test, *t*_10_ = 2.733, *P *=* *0.021) was above chance level.

With data collapsed across all three stimulus types, there was no effect of genotype on distance travelled during either the sample phase (*F*_1,19_ = 1.824, *P *=* *0.193) or the test phase (*F*_1,19_ = 0.471, *P *=* *0.501). Likewise, total stimulus contact time was no different between *Dao* ^+/+^ and *Dao* ^−/−^ mice during either the sample phase (*F*_1,19_ = 0.045, *P *=* *0.834) or test phase (*F*_1,19_ = 0.357, *P *=* *0.557). Hence, the superior recognition memory performance of *Dao* ^−/−^ mice did not result from increased exploration of the experimental stimuli during the sample phase. Finally, genotype had no effect on locomotor activity during habituation trials, when there were no experimental stimuli in the arena (see Supporting Information).

#### T‐maze spontaneous alternation

In the T‐maze, *Dao* ^−/−^ mice demonstrated enhanced spontaneous alternation relative to *Dao* ^+/+^ mice (*F*_1,44_ = 4.244, *P *=* *0.045, Fig. 2E). Performance was above chance level (50%) in both *Dao* ^+/+^ mice (Student's *t*‐test, *t*_23_ = 2.532, *P *=* *0.019) and *Dao* ^−/−^ mice (Student's *t*‐test, *t*_23_ = 5.784, *P *<* *0.001). Genotype had no effect on trial completion (*F*_1,44_ = 1.431, *P *=* *0.238).

### Anxiety

#### Open field test

**Figure 3:**
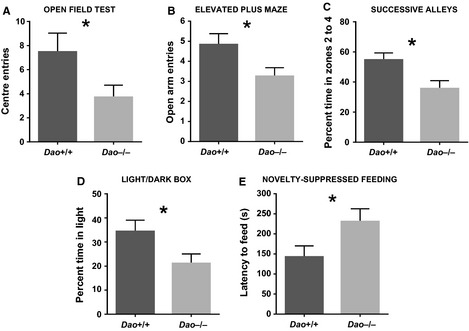
Evidence of heightened anxiety in *Dao* ^−/−^ mice. (A) *Dao* ^−/−^ mice made fewer centre entries than *Dao* ^+/+^ mice in the open field test. (B) *Dao* ^−/−^ mice made fewer open‐arm entries than *Dao* ^+/+^ mice in the elevated plus maze. (C) *Dao* ^−/−^ mice spent less time than *Dao* ^+/+^ mice in the exposed zones (zones 2–4) of the successive alleys apparatus. (D) *Dao* ^−/−^ mice spent less time than *Dao* ^+/+^ mice in the light compartment of the light/dark box. (E) *Dao* ^−/−^ mice took longer than *Dao* ^+/+^ mice to begin feeding in a test of novelty‐suppressed feeding (note that latency to first contact with the food was subtracted from absolute latency to begin feeding, to control for differences in locomotor activity). *P ≤ 0.05. Error bars depict the SEM.

The *Dao* ^−/−^ mice showed elevated anxiety in the open field test. They made fewer centre entries than *Dao* ^+/+^ mice (*F*_1,34_ = 4.765, *P *=* *0.036, Fig. 3A), despite the fact that there was no difference in overall locomotor activity, as measured by total distance travelled (*F*_1,34_ = 0.035, *P *=* *0.853). Percent distance travelled in the centre of the arena was also lower in *Dao* ^−/−^ than *Dao* ^+/+^ mice, although this trend did not quite reach statistical significance (*F*_1,34_ = 3.524, *P *=* *0.069). Genotype had no effect on percent time spent in the centre (*F*_1,34_ = 1.728, *P *=* *0.197), or on latency to the first centre entry (*F*_1,34_ = 1.500, *P *=* *0.229).

#### Elevated plus maze

The *Dao* ^−/−^ mice also demonstrated increased anxiety in the elevated plus maze. They made fewer open‐arm entries than *Dao* ^+/+^ mice (*F*_1,44_ = 5.101, *P *=* *0.029, Fig. 3B), whereas there was no difference in closed‐arm entries (*F*_1,44_ = 0.003, *P *=* *0.958). *Dao* ^−/−^ mice also exhibited a non‐significant tendency to spend less time than *Dao* ^+/+^ mice in the open arms of the maze (*F*_1,44_ = 3.203, *P *=* *0.080). Genotype had no effect on latency to the first open‐arm entry (*F*_1,44_ = 0.303, *P *=* *0.585), or on total distance travelled (*F*_1,44_ = 3.015, *P *=* *0.090).

#### Successive alleys

The *Dao* ^−/−^ mice displayed heightened anxiety in the successive alleys test. They spent less time than *Dao* ^+/+^ mice in the exposed, open zones of the apparatus (i.e. zones 2–4) (*F*_1,20_ = 7.072, *P *=* *0.015, Fig. 3C). *Dao* ^−/−^ mice also made fewer entries to zone 3 than *Dao* ^+/+^ mice (*F*_1,20_ = 4.370, *P *=* *0.050). No mice of either genotype entered zone 4. Genotype had no effect on total distance travelled (*F*_1,20_ = 1.030, *P *=* *0.322).

#### Light/dark box

The *Dao* ^−/−^ mice also showed elevated anxiety in the light/dark box. They spent less time in the light compartment than *Dao* ^+/+^ mice (*F*_1,32_ = 4.469, *P *=* *0.042, Fig. 3D), although genotype had no effect on the number of light/dark crossings (*F*_1,32_ = 2.476, *P *=* *0.125), or on latency to the first crossing (*F*_1,32_ = 1.993, *P *=* *0.168).

#### Novelty‐suppressed feeding

The *Dao* ^−/−^ mice demonstrated heightened anxiety in the novelty‐suppressed feeding test. Importantly, genotype had no effect on latency to first contact with the novel food (*F*_1,20_ = 0.014, *P *=* *0.906), but *Dao* ^−/−^ mice took longer to begin feeding relative to *Dao* ^+/+^ mice (*F*_1,20_ = 5.134, *P *=* *0.035, Fig. 3E). Interestingly, this effect was driven exclusively by female *Dao* ^−/−^ mice. Amongst males, genotype had no effect on feeding latency (*F*_1,10_ = 0.014, *P *=* *0.908), but amongst females, feeding latency was greater in *Dao* ^−/−^ than *Dao* ^+/+^ mice (*F*_1,10_ = 11.640, *P *=* *0.007). Hence, there was a genotype‐by‐sex interaction for this variable (*F*_1,20_ = 5.937, *P *=* *0.024). By contrast, genotype‐by‐sex interactions were not evident in any of the other experiments described in this article. All simple main effects of sex are described in the Supporting Information.

### Circadian rhythms and sleep

#### Wheel‐running (circadian) analyses

**Figure 4:**
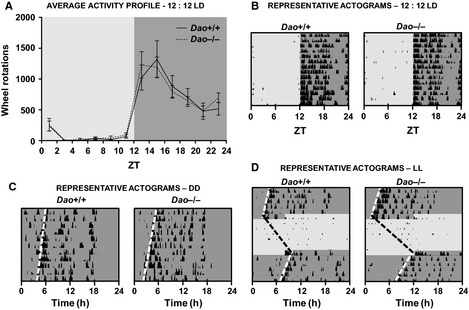
No evidence of circadian rhythm disruption in *Dao* ^−/−^ mice. (A) Average activity profiles for *Dao* ^+/+^ and *Dao* ^−/−^ mice during a 12 : 12 LD cycle at 100 lux. Activity profiles are based on 14 consecutive days of wheel‐running data. Data are presented in 2 h time bins. Representative actograms of *Dao* ^+/+^ and *Dao* ^−/−^ mice during 12 : 12 LD (B), DD (C), and LL (D). Each row depicts a single 24 h period. The light and dark grey shading corresponds to periods of (100 lux) light and dark, respectively. The black bars denote periods of wheel‐running activity, presented in 6 min epochs. The height of the bars corresponds to the number of wheel rotations within each epoch. Error bars depict the SEM.

Spontaneous locomotor activity in wheel‐running cages was unaltered in *Dao* ^−/−^ mice; genotype had no effect on average daily activity counts under DD (*F*_1,10_ = 1.484, *P *=* *0.251), LL (*F*_1,10_ = 3.122, *P *=* *0.108) or 12 : 12 LD (*F*_1,22_ = 0.033, *P *=* *0.858). (Note that mean values for all circadian analyses are included in Table 1 and Table S2.) Furthermore, the temporal distribution of activity over the 24 h day was almost identical in *Dao* ^+/+^ and *Dao* ^−/−^ mice under 12 : 12 LD (Fig. 4A).

**Table 1:** Descriptive statistics for selected wheel‐running (circadian) parameters

Parameter	*Dao* ^+/+^ (mean ± SEM)	*Dao* ^−/−^ (mean ± SEM)	*P*‐value
Daily activity (wheel rotations)	5289 ± 1023	5504 ± 603	0.858
Daily activity bouts	5.6 ± 0.6	6.8 ± 0.4	0.110
Activity bout duration (min)	48 ± 7	52 ± 6	0.674
Intradaily variation (AU)	1.53 ± 0.08	1.66 ± 0.09	0.294
Onset tau (h)	24.02 ± 0.03	24.00 ± 0.01	0.347
Onset tau error (h)	0.82 ± 0.37	0.28 ± 0.10	0.172
Alpha (h)	10.98 ± 0.81	12.26 ± 0.32	0.156
Chi‐square periodogram amplitude (AU)	1177 ± 100	1198 ± 91	0.883
Phase of entrainment (h)	0.074 ± 0.022	0.086 ± 0.021	0.691
Relative light phase activity (%)	4.8 ± 1.0	6.1 ± 1.9	0.527
Interdaily stability (AU)	0.63 ± 0.06	0.71 ± 0.05	0.326

Statistics are derived from 14 consecutive days of recording under a 12 : 12 LD cycle at 100 lux. Sample size is 12 for *Dao* ^+/+^ mice and 12 for *Dao* ^−/−^ mice in all analyses. AU, arbitrary units.

There was also no evidence of altered circadian rhythm fragmentation in *Dao* ^−/−^ mice; genotype had no effect on average daily activity bouts in either DD (*F*_1,10_ = 0.991, *P *=* *0.343), LL (*F*_1,10_ = 4.140, *P *=* *0.069) or 12 : 12 LD (*F*_1,22_ = 2.780, *P *=* *0.110). Likewise, there was no difference in average bout duration in DD (*F*_1,10_ = 0.726, *P *=* *0.414), LL (*F*_1,10_ = 2.253, *P *=* *0.164) or 12 : 12 LD (*F*_1,22_ = 0.181, *P *=* *0.674). Intradaily variation (another measure of circadian fragmentation) was also unaffected by genotype in 12 : 12 LD (*F*_1,22_ = 1.158, *P *=* *0.294).

The *Dao* ^+/+^ and *Dao* ^−/−^ mice were equally able to entrain to a standard 12 : 12 LD cycle (Fig. 4A and B). In 12 : 12 LD, genotype had no effect on onset tau, onset tau error, alpha, chi‐square periodogram amplitude, phase of entrainment, relative light phase activity, or interdaily stability (see Table 1). Similarly, *Dao* ^+/+^ and *Dao* ^−/−^ mice were equally able to adjust to a shift in the light/dark cycle. Following a 6 h phase advance in 12 : 12 LD, genotype had no effect on the number of days required for re‐entrainment (*F*_1,10_ = 0.031, *P *=* *0.864).

Finally, there was no evidence of altered photosensitivity in *Dao* ^−/−^ mice. When exposed to a type II phase‐shifting light pulse from ZT16 to ZT17, percent activity suppression (negative masking) was similar in *Dao* ^+/+^ and *Dao* ^−/−^ mice (*F*_1,9_ = 1.354, *P *=* *0.274), as was the magnitude of the phase delay induced by the light pulse (*F*_1,7_ = 0.318, *P *=* *0.590). Likewise, genotype had no effect on free‐running period length in either DD (*F*_1,10_ = 0.330, *P *=* *0.579, Fig. 4C) or LL (*F*_1,10_ = 1.160, *P *=* *0.307; Fig. 4D).

#### Video‐tracking (sleep) analyses

**Figure 5:**
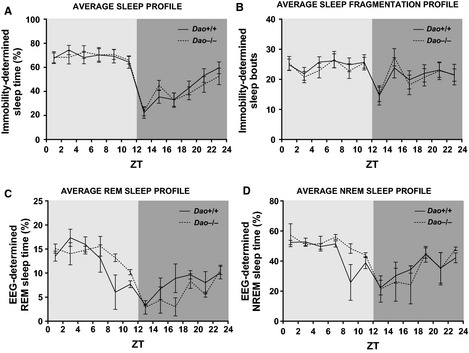
No evidence of sleep disruption in *Dao* ^−/−^ mice. (A) Average immobility‐determined sleep profiles for *Dao* ^+/+^ and *Dao* ^−/−^ mice during a 12 : 12 LD cycle at 100 lux. (B) Average temporal distribution of immobility‐determined sleep bouts in *Dao* ^+/+^ and *Dao* ^−/−^ mice during 12 : 12 LD. A sleep bout was defined as a period of immobility of at least 40 s. Average EEG‐determined REM sleep profiles (C) and NREM sleep profiles (D) for *Dao* ^+/+^ and *Dao* ^−/−^ mice during 12 : 12 LD. The increased variance at ZT8‐10 was due to one *Dao* ^+/+^ mouse sleeping for only 2.7% of this time bin. All plots are based on 24 h of data, presented in 2 h time bins. Error bars depict the SEM.

These analyses revealed no evidence of altered sleep in *Dao* ^−/−^ mice. As expected, there was a significant main effect of time bin on sleep time, sleep bouts and distance travelled (*P*‐values < 0.001), but genotype had no effect on sleep time (*F*_1,22_ = 0.028, *P *=* *0.868), sleep bouts (*F*_1,22_ = 0.055, *P *=* *0.816), or distance travelled (*F*_1,22_ = 0.210, *P *=* *0.652). With the light and dark phases considered in isolation, these variables still did not vary according to genotype (see Table 2). Moreover, the temporal distribution of immobility‐determined sleep was broadly consistent between *Dao* ^+/+^ and *Dao* ^−/−^ mice (Fig. 5A). The same was true of the temporal distribution of sleep bouts (Fig. 5B).

**Table 2:** Descriptive statistics for selected immobility‐determined sleep parameters

Parameter	*Dao* ^+/+^ (mean ± SEM)	*Dao* ^−/−^ (mean ± SEM)	*P*‐value
Total sleep time (%)	55.0 ± 2.4	54.5 ± 2.0	0.868
Light phase sleep time (%)	69.0 ± 3.0	69.4 ± 1.9	0.907
Dark phase sleep time (%)	41.1 ± 2.3	39.6 ± 2.6	0.679
Total sleep bouts	274 ± 11	269 ± 16	0.816
Light phase sleep bouts	149 ± 7	144 ± 6	0.553
Dark phase sleep bouts	125 ± 10	126 ± 13	0.964
Total distance travelled (m)	112.9 ± 16.2	102.9 ± 15.0	0.652
Light phase distance travelled (m)	24.8 ± 4.7	24.5 ± 6.8	0.971
Dark phase distance travelled (m)	88.2 ± 14.3	78.4 ± 9.8	0.577

Statistics are derived from 24 h of recording during a 12 : 12 LD cycle at 100 lux. Sample size is 12 for *Dao* ^+/+^ mice and 12 for *Dao* ^−/−^ mice in all analyses.

#### Electroencephalography‐based sleep analyses

Immobility‐determined sleep analyses cannot differentiate REM and NREM sleep, so EEG‐based sleep analyses were also performed. Again, we found no evidence of altered sleep in *Dao* ^−/−^ mice. As expected, there was a significant main effect of time bin on total sleep, REM sleep and NREM sleep time (*P*‐values < 0.001), but genotype had no effect on total sleep (*F*_1,4_ = 1.472, *P *=* *0.292), REM sleep (*F*_1,4_ = 0.131, *P *=* *0.735), or NREM sleep time (*F*_1,4_ = 2.658, *P *=* *0.178). These variables still did not vary according to genotype when the light and dark phases were considered separately (see Table 3). Furthermore, the temporal distribution of EEG‐determined sleep was broadly consistent between *Dao* ^+/+^ and *Dao* ^−/−^ mice (Fig. S3). The same was true of the temporal distribution of REM sleep (Fig. 5C) and NREM sleep (Fig. 5D).

**Table 3:** Descriptive statistics for selected electroencephalography‐determined sleep parameters

Parameter	*Dao* ^+/+^ (mean ± SEM)	*Dao* ^−/−^ (mean ± SEM)	*P*‐value
Total sleep time (%)	50.0 ± 1.4	52.1 ± 1.1	0.292
Light phase sleep time (%)	57.4 ± 4.3	65.1 ± 3.0	0.218
Dark phase sleep time (%)	42.6 ± 4.1	39.2 ± 4.7	0.606
Total REM sleep time (%)	10.0 ± 0.5	9.8 ± 0.4	0.735
Light phase REM sleep time (%)	12.3 ± 1.7	13.8 ± 0.7	0.440
Dark phase REM sleep time (%)	7.8 ± 1.1	5.8 ± 0.7	0.204
Total NREM sleep time (%)	40.0 ± 0.9	42.3 ± 1.2	0.178
Light phase NREM sleep time (%)	45.1 ± 2.8	51.3 ± 3.0	0.209
Dark phase NREM sleep time (%)	34.9 ± 3.0	33.4 ± 4.1	0.785

Statistics are derived from 24 h of recording during a 12 : 12 LD cycle at 100 lux. Sample size is 3 for *Dao* ^+/+^ mice and 3 for *Dao* ^−/−^ mice in all analyses.

## Discussion

The present study investigated the behavioural characteristics of *Dao* knockout (*Dao* ^−/−^) mice and their wildtype (*Dao* ^+/+^) littermates. We report three main findings. Firstly, *Dao* ^−/−^ mice displayed improved short‐term memory performance in both spatial and non‐spatial tasks; they demonstrated enhanced spatial recognition memory performance, improved odour recognition memory performance, and enhanced spontaneous alternation in the T‐maze. Secondly, heightened anxiety‐like behaviour was observed in *Dao* ^−/−^ mice in five tests of approach/avoidance conflict: the open field test, elevated plus maze, successive alleys, light/dark box and novelty‐suppressed feeding. This phenotype was not an artefact of altered locomotor activity; *Dao* ^−/−^ mice were no more or less active than *Dao* ^+/+^ mice in any of the anxiety tests in which distance data were recorded. Likewise, genotype had no effect on distance travelled in a standard laboratory test of spontaneous locomotor activity, or in any of our sleep or circadian analyses. Thirdly, *Dao* ^−/−^ mice showed no evidence of sleep or circadian rhythm disruption.

### Phenotypic comparison with *Dao* mutants and relevant pharmacological models

The ddY/*Dao* ^−^ mouse is a naturally‐occurring mutant mouse that lacks DAO activity due to a point mutation in the gene (Konno & Yasumura, 1983; Konno *et al*., 1991), while the *Dao1* ^*G181R*^ mouse was created by transferring the same mutation from a ddY to a C57BL/6J background (Labrie *et al*., 2009a). Behavioural phenotyping of these mutants has focused predominantly on tests of long‐term spatial memory; ddY/*Dao* ^−^ mice show enhanced performance in the Barnes maze (Zhang *et al*., 2011) and improved acquisition in the MWM (Maekawa *et al*., 2005), whereas *Dao1* ^*G181R*^ mice display enhanced MWM reversal learning (Labrie *et al*., 2009b). Enhanced long‐term memory performance has also been observed in wildtype rodents after the administration of d‐serine or DAO inhibitors (Duffy *et al*., 2008; Hashimoto *et al*., 2008; Karasawa *et al*., 2008; Smith *et al*., 2009; Bado *et al*., 2011; Hopkins *et al*., 2013). *Dao* ^−/−^ mice demonstrate a complex behavioural phenotype in the MWM (D. Pritchett, A.M. Taylor, S.J. Engle, N.J. Brandon, T. Sharp, R.G. Foster, P.J. Harrison, S.N. Peirson and D.M. Bannerman, in preparation), although a full description of this phenotype is beyond the scope of the current article. Crucially, the present findings represent the first demonstration of enhanced *short‐term* memory performance in a genetic mouse model lacking DAO activity. Our T‐maze data echo the observation of enhanced T‐maze rewarded alternation in wildtype mice after d‐serine administration (Bado *et al*., 2011).

Our anxiety results are broadly consistent with the *Dao* mutant literature; elevated anxiety‐like behaviour has been reported in both ddY/*Dao* ^−^ mice and *Dao1* ^*G181R*^ mice in the elevated plus maze and open field test, particularly in females (Labrie *et al*., 2009a). Another study failed to observe increased anxiety in ddY/*Dao* ^−^ mice, but female mice were not included in these experiments (Zhang *et al*., 2011). In the present study, elevated anxiety‐like behaviour was apparent in male and female *Dao* ^−/−^ mice in the open field test, elevated plus maze, successive alleys and light/dark box, but was only apparent in female *Dao* ^−/−^ mice in the novelty‐suppressed feeding task. Our anxiety data are also consistent with the pharmacological literature; wildtype mice (of both sexes) display heightened anxiety‐like behaviour in the open field test following d‐serine administration (Labrie *et al*., 2009a).

The behavioural consequences of DAO inactivity appear relatively robust, given that heightened anxiety and enhanced memory performance have now been witnessed across three different genetic constructs. Whereas ddY/*Dao* ^−^ mice (and presumably *Dao1* ^*G181R*^ mice) show normal expression of a DAO enzyme rendered inactive by a point mutation (Konno & Yasumura, 1983; Konno *et al*., 1991; Almond *et al*., 2006), *Dao* ^−/−^ mice show no detectable expression of *Dao* mRNA or DAO protein (Schweimer *et al*., 2014). Our use of a constitutive knockout thereby eliminates the potential influence of a widely expressed (albeit catalytically inactive) DAO enzyme.

Moreover, there is phenotypic overlap between the models despite the use of different background strains (ddY, C57BL/6J and 129SvEv), each of which differs in terms of baseline anxiety and cognitive performance. 129 substrains, for example, are characterized by increased anxiety and impaired memory performance relative to other inbred strains (Voikar *et al*., 2001; Rodgers *et al*., 2002; Brooks *et al*., 2005; Koike *et al*., 2006; Molenhuis *et al*., 2014). Our use of the 129SvEv strain may explain the generally poor recognition memory performance of *Dao* ^+/+^ mice in the present study, which did not exceed chance in either odour or spatial recognition trials. Nonetheless, the performance of *Dao* ^+/+^ mice did exceed chance in the T‐maze spontaneous alternation task; hence, the significant advantage of *Dao* ^−/−^ mice in this task cannot be attributed to the poor performance of controls.

### Mechanistic basis of altered memory performance and anxiety in *Dao* ^−/−^ mice

d‐amino acid oxidase is traditionally seen as a hindbrain enzyme (Horiike *et al*., 1994; Schell *et al*., 1995), and indeed, d‐serine is not increased in the prefrontal cortex of ddY/*Dao* ^−^ mice (see Yamanaka *et al*., 2012), *Dao1* ^*G181R*^ mice (Labrie *et al*., 2009b), or *Dao* ^−/−^ mice (Rais *et al*., 2012). The behavioural phenotypes of these models may therefore reflect the absence of DAO activity in other brain regions, such as the cerebellum, wherein d‐serine levels are markedly increased in all three models (see Labrie *et al*., 2009b; Rais *et al*., 2012; Yamanaka *et al*., 2012). d‐serine is also moderately elevated in the hippocampus of *Dao1* ^*G181R*^ mice (Labrie *et al*., 2009b) and *Dao* ^−/−^ mice (Pfizer Inc., unpublished data). Conceivably, altered hippocampal function could underlie aspects of both the anxiety and spatial memory phenotypes of *Dao* ^−/−^ mice, given that the ventral and dorsal hippocampus make dissociable contributions to anxiety and memory function, respectively (see Bannerman *et al*., 2014).

Despite this evidence, it is not certain that elevated d‐serine is responsible for the behavioural phenotypes of mice lacking functional DAO. Although d‐serine is the most abundant substrate of DAO in the mammalian brain, DAO also metabolizes d‐alanine, another NMDAR co‐agonist (Tanii *et al*., 1994; Sakata *et al*., 1999). d‐alanine levels are increased uniformly throughout the brain in ddY/*Dao* ^−^ mice (see Yamanaka *et al*., 2012), whereas d‐alanine levels in *Dao1* ^*G181R*^ and *Dao* ^−/−^ mice remain to be elucidated.

As a constitutive knockout model, the observed behavioural phenotype of the *Dao* ^−/−^ mouse could be a consequence of altered neurodevelopment. Consistent with this suggestion, there is evidence that implicates DAO in cerebellar development (see Yamanaka *et al*., 2012). Having said this, several d‐serine ‐related genes (e.g. *Asc‐1*,* GlyT1*,* Srr* and *Grin1*) show unaltered expression in the brains of ddY/*Dao* ^−^ mice and cerebellar DAO knockdown mice (see Yamanaka *et al*., 2012). Moreover, the increased anxiety‐like behaviour and enhanced spontaneous alternation of *Dao* ^−/−^ mice closely resemble the increased anxiety‐like behaviour and enhanced rewarded alternation of wildtype mice following d‐serine administration (Labrie *et al*., 2009a; Bado *et al*., 2011). These pharmacological data infer, but do not prove, that the altered behaviour of the *Dao* ^−/−^ mouse results from an increase in brain d‐serine.

It is known that *Dao* is expressed in the ventral tegmental area (Betts *et al*., 2014), while a recent study observed increased burst‐firing of ventral tegmental area dopaminergic neurons in *Dao* ^−/−^ mice (Schweimer *et al*., 2014). Altered dopaminergic function in the ventral tegmental area might contribute to the enhanced recognition memory performance of *Dao* ^−/−^ mice, given that a recent *in vivo* calcium imaging study found that the activity of dopaminergic ventral tegmental area neurons was predictive of object recognition memory performance in mice (Gunaydin *et al*., 2014).

### Relationship to other mouse models of altered *N*‐methyl‐d‐aspartate receptor co‐agonism

Our data fit neatly into a wider preclinical literature concerning the relationship between NMDAR co‐agonism and behaviour. Serine racemase (SRR), for example, is an enzyme that catalyses the synthesis of d‐serine (Wolosker *et al*., 1999). Unlike mouse models of DAO inactivation/deletion, mouse models of SRR inactivation/deletion exhibit *reduced* levels of brain d‐serine and demonstrate *impaired* MWM and spatial recognition memory performance (Basu *et al*., 2009; Labrie *et al*., 2009c). Impaired MWM and spatial recognition memory performance have also been observed in *Grin1* ^*D481N*^ mutant mice (Kew *et al*., 2000; Duffy *et al*., 2008; Labrie *et al*., 2008), which possess NMDARs with a fivefold reduction in d‐serine affinity (Kew *et al*., 2000). Significantly, the cognitive deficits of both *Srr* ^*Y269*^ mutant mice and *Grin1* ^*D481N*^ mutant mice are reversible by d‐serine administration (Duffy *et al*., 2008; Labrie *et al*., 2008, 2009c). In contrast to these models, MWM and object recognition memory performance are *enhanced* in glycine transporter 1 (GlyT1)‐deficient mice (Mohler *et al*., 2011). Glycine, like d‐serine, is an NMDAR co‐agonist, and GlyT1 is the principal regulator of glycine concentration at NMDAR‐containing synapses (Mohler *et al*., 2011). Taken together, these data provide strong evidence of a positive association between NMDAR co‐agonism and memory performance in mice. *Grin1* ^*D481N*^ mice also demonstrate a reduced anxiety phenotype in the elevated plus maze, open field test and object neophobia task, which again can be reversed with d‐serine (Labrie *et al*., 2009a). Hence, there also seems to be a positive correlation between NMDAR co‐agonism and anxiety levels.

### No sleep or circadian rhythm disruption in *Dao* ^−/−^ mice

In rodents, there is evidence of a bidirectional relationship between anxiety and sleep and circadian function. In one direction, sleep deprivation alters behaviour in standard tests of anxiety such as the elevated plus maze (e.g. Silva *et al*., 2004), while in the other, exposure to environmental stressors impacts on subsequent sleep, particularly REM sleep (Koehl *et al*., 2006; Tang *et al*., 2007; Pawlyk *et al*., 2008). In addition, mouse models of high trait anxiety demonstrate sleep irregularities such as increased dark phase sleep and increased sleep fragmentation in 12 : 12 LD (Jakubcakova *et al*., 2012). They also display circadian abnormalities such as increased activity fragmentation in 12 : 12 LD and DD, and lengthened tau in DD (Griesauer *et al*., 2014). In the light of these findings, it was somewhat surprising to find no evidence of sleep or circadian rhythm disruption in *Dao* ^−/−^ mice. Instead, our data indicate that altered sleep or circadian rhythms are neither a cause nor a consequence of the elevated anxiety seen in *Dao* ^−/−^ mice. More generally, our data reveal that sleep and circadian rhythm disruption are not inevitable correlates of heightened anxiety in mice.

Another reason to evaluate circadian function in *Dao* ^−/−^ mice was that NMDARs play a critical role at the junction between the retinohypothalamic tract and suprachiasmatic nucleus, the brain's principal circadian pacemaker (Moore, 1973; Moore & Klein, 1974). Previous studies have shown that the NMDAR antagonists MK‐801 and 3(2‐carboxypiperazin‐4‐yl)‐propyl‐1‐phosphonic acid (CPP) block the phase‐shifting effects of light on the wheel‐running rhythms of mice and hamsters (Colwell *et al*., 1991; Colwell & Menaker, 1992). Based on this evidence, it was considered that *Dao* ^−/−^ mice might demonstrate heightened sensitivity to light (i.e. increased phase‐shifting and negative masking in response to nocturnal light pulses, and increased period lengthening in LL). We found no evidence to support this hypothesis, suggesting that DAO does not modulate glutamatergic neurotransmission at retinohypothalamic tract–suprachiasmatic nucleus synapses.

d‐amino acid oxidase also has an established physiological role in the rodent retina, where it modulates NMDAR‐mediated glutamatergic neurotransmission between bipolar cells and retinal ganglion cells (Stevens *et al*., 2003; Gustafson *et al*., 2007, 2013). However, although DAO may be active in the retina, the absence of a circadian phenotype in *Dao* ^−/−^ mice indicates that DAO is not essential for non‐image‐forming visual function. This is perhaps unsurprising given that circadian photoentrainment is resistant to dramatic retinal changes, including the complete ablation of rods and cones, which renders mice visually blind (Freedman *et al*., 1999).

### Clinical implications

Our findings are of particular relevance to schizophrenia, a disorder in which memory impairments are both debilitating and resistant to existing treatments (Geyer & Tamminga, 2004). There is moderate evidence that *Dao* is a susceptibility gene for the disorder (Chumakov *et al*., 2002; Allen *et al*., 2008; Shi *et al*., 2008; Sun *et al*., 2008), while *Dao* expression and DAO activity are elevated in the brains of patients with schizophrenia (Kapoor *et al*., 2006; Verrall *et al*., 2007; Burnet *et al*., 2008; Madeira *et al*., 2008; Habl *et al*., 2009; Ono *et al*., 2009). Conversely, d‐serine concentration is reduced in patients' serum and cerebrospinal fluid (Hashimoto *et al*., 2003, 2005; Yamada *et al*., 2005; Bendikov *et al*., 2007; Brouwer *et al*., 2013). Given that d‐serine is an NMDAR co‐agonist, the overactivity of DAO (and resulting insufficiency of d‐serine) could contribute to the NMDAR hypofunction proposed to exist in schizophrenia (Olney *et al*., 1999; Kantrowitz & Javitt, 2010; Marek *et al*., 2010; Verrall *et al*., 2010; Coyle, 2012; Labrie *et al*., 2012).

Consequently, DAO inhibitors are currently under development for the treatment of schizophrenia (Smith *et al*., 2010; Ferraris & Tsukamoto, 2011; Sacchi *et al*., 2013), and there is already some evidence that testifies to their efficacy. In patients with schizophrenia, adjunctive treatment with the DAO inhibitor sodium benzoate was effective against a range of symptom domains, including cognition, in a small 6 week randomized clinical trial (Lane *et al*., 2013). Interestingly, no emergent anxiety symptoms were observed in this study, or in a trial of sodium benzoate in dementia (Lin *et al*., 2014). Although this could reflect species differences, it will remain important to assess anxiety in future clinical studies; it is possible that heightened anxiety could result from chronic but not acute DAO inhibition. d‐serine has also been administered to patients with schizophrenia, with moderate success (e.g. Kantrowitz *et al*., 2010), although there are concerns about possible side‐effects such as nephrotoxicity (see Ganote *et al*., 1974; Carone & Ganote, 1975; Kantrowitz *et al*., 2010).

## Summary

Our findings add to a considerable body of preclinical evidence, both genetic and pharmacological, linking altered DAO function with a range of behavioural changes (e.g. Maekawa *et al*., 2005; Labrie *et al*., 2009a,b; Zhang *et al*., 2011; Hopkins *et al*., 2013). It is now apparent that the inactivation or deletion of DAO can both heighten anxiety and improve memory performance in multiple domains, including both long‐term and short‐term memory tasks, of both a spatial and non‐spatial nature. The underlying mechanisms remain to be elucidated, but the ubiquity of the phenotype hints at global changes in information processing and/or memory encoding and retrieval.

## Supporting information

Fig. S1. No evidence of altered spontaneous locomotor activity in *Dao* ^−/−^ mice.
Fig. S2. Analysis of recognition memory performance in terms of contact time with the novel and familiar stimuli during the test phase.
Fig. S3. Average EEG‐determined sleep profiles for *Dao* ^+/+^ and *Dao* ^−/−^ mice during a 12 : 12 h light/dark (12 : 12 LD) cycle at 100 lux.
Table S1. Summary of cohorts used for sleep, circadian and behavioural testing.
Table S2. Descriptive statistics for selected wheel‐running (circadian) parameters.
