# Supplementary material for: d‐amino acid oxidase knockout (Dao −/−) mice show enhanced short‐term memory performance and heightened anxiety, but no sleep or circadian rhythm disruption
Source: Eur J Neurosci. 2015 Mar 27;41(9):1167–79. doi: 10.1111/ejn.12880 (PMC4744680; doi:10.1111/ejn.12880)
Supplement: Supplementary file 1 — Fig. S1. No evidence of altered spontaneous locomotor activity in Dao −/− mice. Fig. S2. Analysis of recognition memory performance in terms of contact time with the novel and familiar stimuli during the test phase. Fig. S3. Average EEG‐determined sleep profiles for Dao +/+ and Dao −/− mice during a 12 : 12 h light/dark (12 : 12 LD) cycle at 100 lux. Table S1. Summary of cohorts used for sleep, circadian and behavioural testing. Table S2. Descriptive statistics for selected wheel‐running (circadian) parameters. [file EJN-41-1167-s001.pdf]

Supplementary information

Methods: Animals

Cohorts A1 and A2 were group-housed (5-7 animals per cage). Cohorts C1, C2 and D were singly-housed to enable the analysis of sleep and circadian rhythms in individual animals. Cohorts B1 and B2 were singly-housed to allow direct comparison with cohorts C1, C2 and D.

Cohort	Housing	Composition	Behavioural Tests (in chronological order)	Time of Test
A1	Group	2 × *Dao*$^{+/+}$ ♂; 7 × *Dao*$^{+/+}$ ♀;<br>6 × *Dao*$^{-/-}$ ♀	Open field test<br><br>Light/dark box	ZT13 – ZT18<br><br>ZT13 – ZT18
A2	Group	5 × *Dao*$^{+/+}$ ♂; 7 × *Dao*$^{+/+}$ ♀;<br>6 × *Dao*$^{-/-}$ ♂; 5 × *Dao*$^{-/-}$ ♀	Open field test<br><br>Light/dark box<br><br>Spontaneous recognition memory	ZT13 – ZT18<br><br>ZT13 – ZT18<br><br>ZT19 – ZT23
B1	Single	6 × *Dao*$^{+/+}$ ♂; 6 × *Dao*$^{+/+}$ ♀;<br>6 × *Dao*$^{-/-}$ ♂; 6 × *Dao*$^{-/-}$ ♀	Elevated plus maze<br><br>Successive alleys<br><br>Novelty-suppressed feeding<br><br>Spontaneous locomotor activity<br><br>T-maze spontaneous alternation	ZT3 – ZT7<br><br>ZT3 – ZT7<br><br>ZT1 – ZT4<br><br>ZT3 – ZT7<br><br>ZT3 – ZT4 and ZT7 – ZT8
B2	Single	6 × *Dao*$^{+/+}$ ♂; 6 × *Dao*$^{+/+}$ ♀;<br>6 × *Dao*$^{-/-}$ ♂; 6 × *Dao*$^{-/-}$ ♀	Elevated plus maze<br><br>T-maze spontaneous alternation	ZT3 – ZT7<br><br>ZT3 – ZT4 and ZT7 – ZT8
C1	Single	6 × *Dao*$^{+/+}$ ♂; 6 × *Dao*$^{-/-}$ ♂	Wheel-running analyses (12:12 LD; 6 h phase advance; DD; LL; type II light pulse) and video-tracking analyses (12:12 LD only)	Continuous
C2	Single	6 × *Dao*$^{+/+}$ ♂; 6 × *Dao*$^{-/-}$ ♂	Wheel-running analyses (12:12 LD only) and video-tracking analyses (12:12 LD only)	Continuous
D	Single	3 × *Dao*$^{+/+}$ ♂; 3 × *Dao*$^{-/-}$ ♂	EEG-based sleep analyses	Continuous

SUPPLEMENTARY TABLE 1. Summary of cohorts used for sleep, circadian and behavioural testing. ZT = zeitgeber time; ZT0 refers to the onset of the light phase, while ZT12 denotes the onset of the dark phase. Mice were at least 2 months old at the onset of all procedures, and no older than 9 months upon their completion.

Methods: Wheel-running (circadian) analyses

Light-tight chambers were maintained at constant temperature (21°C) and humidity (50%). Each light-tight chamber contained 6 cages, with 3 mice of each genotype housed in alternating positions.

Average daily activity counts (i.e. wheel rotations) provided a measure of spontaneous locomotor activity. Circadian rhythm fragmentation was also assessed, given evidence of increased fragmentation in schizophrenia (Wulff *et al.*, 2012). Fragmentation was estimated by calculating the average number of activity bouts (maximum gap: 18 min; threshold: 5 counts/min) per day. Intradaily variation provided another indicator of fragmentation; this non-parametric measure quantifies the frequency and extent of transitions between rest and activity within each 24 h period (Van Someren, 1999).

To assess entrainment during 12:12 LD, multiple parameters were extracted: onset tau (period length), onset tau error, alpha, chi-square periodogram amplitude, phase of entrainment, percent light phase activity, and interdaily stability. Interdaily stability is a non-parametric measure that quantifies the consistency of activity patterns across multiple days (Van Someren *et al.*, 1999). Onset tau (period length) was also computed under constant dark conditions (DD). Period length in constant light (LL) was calculated manually by extrapolation (see Fig. 4D), since activity levels were too low to use the automated method based on activity onsets. Re-entrainment following the 6 h phase advance was scored manually from individual actograms as the number of days taken to re-entrain. Note that interdaily stability and intradaily variation were computed using Actiwatch Activity & Sleep Analysis 7 (Cambridge Neurotechnology, Cambridge, UK).

Cohort C1 was also subjected to a type II phase-shifting light pulse (Aschoff, 1965) – using a previously-described protocol (Albrecht *et al.*, 2001; Jud *et al.*, 2005) – to induce a phase delay in their activity rhythms, providing an indicator of photosensitivity. The 100 lux light pulse was delivered for one hour (ZT16-17) during a standard 12:12 LD cycle. Following the pulse, mice were released into DD for 7 days. The magnitude of the phase delay was quantified by fitting one regression line through 6 consecutive activity onsets preceding the light pulse, and another through 6 consecutive activity onsets following the pulse. The first activity onset following the light pulse was disregarded because of possible transition effects (Jud *et al.*, 2005). The magnitude of the phase delay was calculated as the time difference between the two regression lines on the first day after the light pulse.

Negative masking – the degree of activity suppression induced during the light pulse – was also calculated, yielding an additional measure of photosensitivity (Mrosovsky, 1999). Percent activity suppression was computed by comparing the number of activity counts during the light pulse with the average number of counts during the same time window (ZT16-17) across the previous 3 days.

Results: Spontaneous locomotor activity

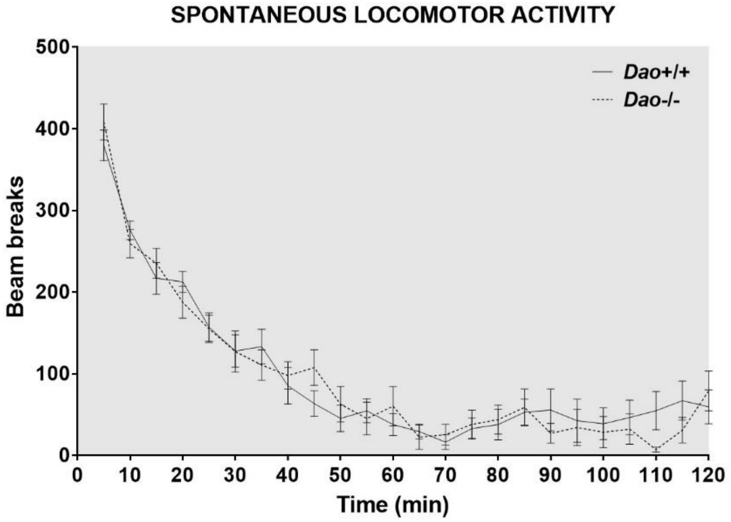

SUPPLEMENTARY FIG. 1. No evidence of altered spontaneous locomotor activity in Dao$^{-/-}$ mice. Activity is presented as the number of beam breaks across twenty-four 5 min time bins.

Results: Spontaneous recognition memory

The enhanced recognition memory performance of *Dao*$^{-/-}$ mice reflects the fact that, during the test phase, *Dao*$^{-/-}$ mice spent more time than *Dao*$^{+/+}$ mice in contact with the novel stimulus, and less time than *Dao*$^{+/+}$ mice in contact with the familiar stimulus (supplementary Fig. 2).

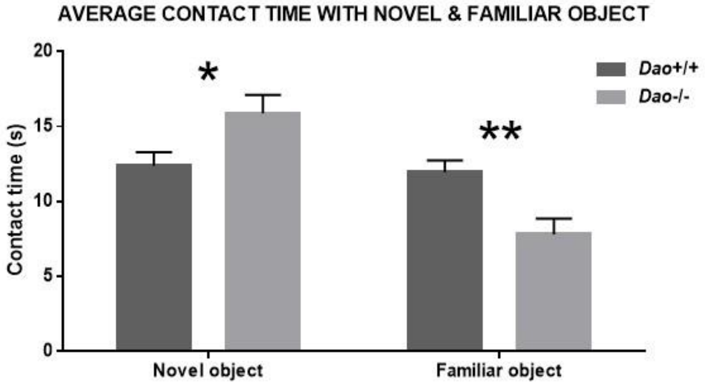

SUPPLEMENTARY FIG. 2. Analysis of recognition memory performance in terms of contact time with the novel and familiar stimuli during the test phase. Dao$^{-/-}$ mice spent more time than Dao$^{+/+}$ mice in contact with the novel stimulus, and less time than Dao$^{+/+}$ mice in contact with the familiar stimulus. This figure depicts average performance across all 6 trials of testing (i.e. 2 object recognition trials, 2 spatial recognition trials and 2 odour recognition trials).

Genotype had no effect on total distance travelled during habituation trials ($F_{1,19} = 2.026$, $P = 0.171$). Similarly, genotype had no effect on the rate of habituation. For example, when distance travelled was split into nine 1 min time bins, there was a main effect of both day ($F_{1,19} = 32.260$, $P = <0.001$) and time bin ($F_{8,152} = 37.558$, $P = <0.001$), reflecting a progressive reduction in activity over time. However, there was no interaction between day and genotype ($F_{1,19} = 0.206$, $P = 0.655$) or time bin and genotype ($F_{8,152} = 0.161$, $P = 0.894$), indicating that *Dao*$^{-/-}$ and *Dao*$^{+/+}$ mice habituated to the arena at a similar rate.

Results: Simple main effects of sex in behavioural tests

Spontaneous locomotor activity

In the test of spontaneous locomotor activity, males made more beam breaks than females during the first 5 min ($F_{1,20}$ = 12.855, P = 0.002), but not over the entire 2 h recording period ($F_{1,20}$ = 0.003, P = 0.959).

Spontaneous recognition memory

In the spontaneous recognition memory test arena, males travelled a greater distance than females during habituation trials ($F_{1,19}$ = 4.908, P = 0.039).

T-maze spontaneous alternation

In the T-maze, there was a main effect of sex on trial completion ($F_{1,44}$ = 5.724, P = 0.021), with males completing fewer trials ($\underline{M}$ = 86.1%) than females ($\underline{M}$ = 97.2%) at the first attempt.

Open field test

In the open field test, females made fewer centre entries than males ($F_{1,34}$ = 8.058, P = 0.008), travelled less distance overall ($F_{1,34}$ = 23.566, P = <0.001), and took longer to make their first centre entry ($F_{1,34}$ = 7.529, P = 0.010). Percent distance travelled in the centre of the arena was also lower in females than males ($F_{1,34}$ = 6.495, P = 0.016).

Light/dark box

Males made more light/dark crossings than females in the light/dark box ($F_{1,32}$ = 5.189, P = 0.030).

Results: Wheel-running (circadian) analyses

Parameter	*Dao*$^{+/+}$ (mean ± SEM)	*Dao*$^{-/-}$ (mean ± SEM)	p-value
Daily activity in DD (wheel rotations)	2854 ± 748 (*n* = 6)	4150 ± 756 (*n* = 6)	0.251
Daily activity bouts in DD	4.4 ± 0.8 (*n* = 6)	5.2 ± 0.2 (*n* = 6)	0.343
Activity bout duration in DD (min)	44 ± 9 (*n* = 6)	54 ± 7 (*n* = 6)	0.414
Onset tau in DD (h)	23.77 ± 0.10 (*n* = 6)	23.69 ± 0.10 (*n* = 6)	0.579
Daily activity in LL (wheel rotations)	61 ± 15 (*n* = 6)	248 ± 105 (*n* = 6)	0.108
Daily activity bouts in LL	0.3 ± 0.2 (*n* = 6)	1.0 ± 0.3 (*n* = 6)	0.069
Activity bout duration in LL (min)	4 ± 2 (*n* = 6)	13 ± 6 (*n* = 6)	0.164
Tau in LL (h)	25.89 ± 0.23 (*n* = 6)	25.50 ± 0.27 (*n* = 6)	0.307
Days to re-entrain after a 6 h phase advance	4.7 ± 0.6 (*n* = 6)	4.5 ± 0.8 (*n* = 6)	0.864
Type II light pulse – negative masking (%)	81.3 ± 9.0 (*n* = 6)	93.3 ± 2.8 (*n* = 5)	0.274
Type II light pulse – phase delay (h)	0.86 ± 0.36 (*n* = 4)	0.63 ± 0.21 (*n* = 5)	0.590

SUPPLEMENTARY TABLE 2. Descriptive statistics for selected wheel-running (circadian) parameters. Statistics are derived from 14 consecutive days of recording under a 12:12 h light/dark (12:12 LD) cycle at 100 lux. Units of measurement and sample sizes are indicated in brackets. SEM = standard error of the mean.

Results: Average EEG-determined sleep profile

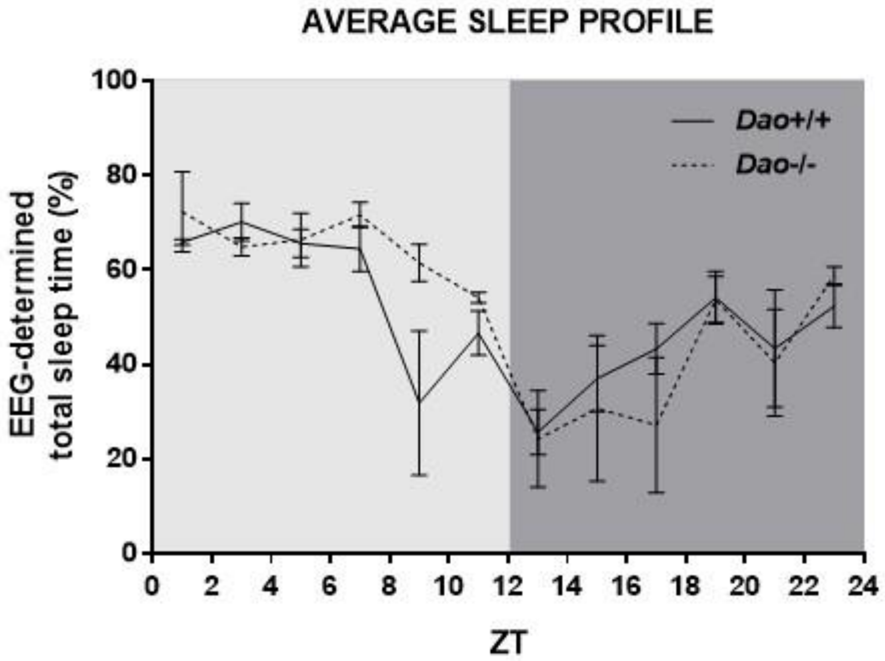

SUPPLEMENTARY FIG. 3. Average EEG-determined sleep profiles for Dao$^{+/+}$ and Dao$^{-/-}$ mice during a 12:12 h light/dark (12:12 LD) cycle at 100 lux. The increased variance at ZT8-10 was due to one Dao$^{+/+}$ mouse sleeping for only 2.7 % of this time bin. This plot is based on 24 h of data, presented in 2 h time bins. ZT = zeitgeber time.
